# Pediatric Hepatocyte Nuclear Factor 1B (*HNF1B*) Disease: Diabetes and Endocrine Manifestations

**DOI:** 10.1155/pedi/4077604

**Published:** 2025-04-27

**Authors:** Meghan Craven, Vaneeta Bamba, Andrew C. Calabria, Sara E. Pinney

**Affiliations:** ^1^ Division of Diabetes and Endocrinology, Department of Pediatrics, Texas Children’s Hospital, Baylor College of Medicine, Houston, Texas, 77030, USA, bcm.edu; ^2^ Division of Endocrinology and Diabetes, Children’s Hospital of Philadelphia, Philadelphia, Pennsylvania, 19104, USA, chop.edu; ^3^ Department of Pediatrics, Perelman School of Medicine, University of Pennsylvania, Philadelphia, Pennsylvania, 19104, USA, upenn.edu

**Keywords:** chronic kidney disease, *HNF1B*, hyperlipidemia, hyperparathyroidism, hypomagnesemia, MODY5, monogenic diabetes, pediatric diabetes, renal cysts, renal dysplasia

## Abstract

**Context:** Mutations in hepatocyte nuclear factor 1B (*HNF1B*) are rare but they are known to cause structural renal disease and diabetes mellitus. There is limited data on pediatric *HNF1B* disease.

**Objective:** To analyze the clinical characteristics of *HNF1B*‐related disease in a cohort of children identified at a single pediatric tertiary medical center, with a specific focus on endocrine‐related disease.

**Methods:** Subjects with *HNF1B* genetic variants were identified from the Children’s Hospital of Philadelphia Atypical Diabetes Registry between 2013 and 2022.

**Results:** Of the 11 pediatric subjects with *HNF1B* mutations or deletions, 7 (64%) initially presented with diabetes, sometimes referred to as MODY5, while 4 (36%) were diagnosed based on family history or a genetic evaluation of renal disease. Only one patient presented with diabetic ketoacidosis, and three presented with diabetic ketosis. Of the four children with *HNF1B* mutations identified by familial mutation analysis or based on renal disease, two developed diabetes during the course of the study. Abnormalities in fasting lipid profiles were common: 10 with triglycerides >90 mg/dL, 5 with LDL‐C >110 mg/dL, 5 with HDL‐C <45, and 7/11 with non‐HDL cholesterol >120 mg/dL. Over half of the subjects had hyperparathyroidism with PTH (>65 pg/mL) and a calcium concentration >9 mg/dL.

**Conclusion:** This case series represents one of the largest pediatric *HNF1B*‐related disease cohorts at a single center. The majority of patients with diabetes presented with clinical features distinct from Type 1 or Type 2 diabetes. Pediatricians should consider genetic testing for *HNF1B* mutations when children are diagnosed with diabetes and have renal abnormalities, hyperlipidemia, and hyperparathyroidism.

## 1. Introduction

Monogenic diabetes is an uncommon form of diabetes mellitus caused by a mutation or deletion of a single gene and is estimated to account for 1%–5% of pediatric diabetes cases [1, 2]. The diagnosis encompasses cases of Maturity‐Onset Diabetes of the Young (MODY) and neonatal diabetes mellitus and represents a wide spectrum of disease, including over 25 genetic subtypes that are often characterized by multiorgan system involvement. Mutations or deletions in *HNF1B* are the underlying genetic etiology of MODY 5 which is associated with health conditions involving the pancreas, kidneys, liver, genital tract, lipid metabolism, calcium homeostasis, and developmental delays along with diabetes mellitus (Table 1). There are many patients who present with symptoms of *HNF1B* disease who do not have diabetes, but as pediatric endocrinologists, the atypical features of *HNF1B* diabetes are often the first presenting symptoms of *HNF1B* disease. While *HNF1B* diabetes is rare, accounting for <1%–6% of monogenic diabetes cases, we propose that careful identification of the unique clinical features of *HNF1B* disease in pediatric diabetes patients should prompt genetic testing to establish a molecular diagnosis [2–4].

**Table 1:** Range of Clinical Characteristics Associated with Pediatric HNF1B Disease.

• Intrauterine growth restriction/SGA birth weight
• Neonatal hyperglycemia
• Neonatal cholestasis
• Pancreatic hypoplasia
• Diabetes mellitus
• Pancreatic exocrine insufficiency
• Renal structural abnormalities
◦ Renal cysts, dysplasia, hydronephrosis, and single kidney
• Chronic kidney disease
• Hypomagnesaemia
• Primary hyperparathyroidism
• Elevated hepatic transaminases
• Hyperlipidemia
• Autism/social pragmatic disorder
• Developmental delays
• Reproductive tract abnormalities

The diagnosis of *HNF1B* diabetes is often confirmed through genetic testing based on early‐onset renal disease. In addition to abnormal development of the kidney and pancreas, patients with *HNF1B* disease have been associated with neurological features, genital tract malformations, and abnormal liver function (Table 1) [5, 6]. This multisystem phenotype is attributable to the HNF1B transcription factor which regulates the expression of many downstream target genes [5–7].

Past studies have found that *HNF1B*‐ disease can result from either a heterozygous microdeletion of chromosome 17q12 region (known as 17q12 recurrent deletion syndrome [ORPHA:261265, OMIM 614527]), or heterozygous *HNF1B* mutations. Further, although these conditions can follow an autosomal dominant inheritance pattern, they can also present as de novo genetic mutations and individuals in families with the same molecular mutation may have widely varying clinical features [5, 8, 9].

Here, we describe summary data characterizing the clinical and biochemical characteristics of 11 pediatric cases with *HNF1B*‐related disease focusing on diabetes and endocrine‐related features. Additionally, we will summarize findings from six other cohorts of individuals with *HNF1B* disease described in the recent literature, focusing on the pediatric presentation of *HNF1B* diabetes, and provide recommendations for diagnosis and treatment of children and adolescents with *HNF1B* disease for the pediatric endocrinologist.

## 2. Methods

### 2.1. Study Population

Subjects were recruited to participate in the Atypical and Monogenic Diabetes Registry at the Children’s Hospital of Philadelphia after they were identified as candidates for an atypical diabetes consultation by their primary diabetes practitioner through a clinical algorithm previously published by Tamaroff et al. [1], or alternatively through external referral if a known mutation associated with monogenic diabetes was identified. The atypical diabetes clinical algorithm selects for patients (1) diagnosed with diabetes <25 years of age (2) without evidence of pancreatic islet autoimmunity (GAD 65, ICA512, IAA, and ZNT8), and (3) either a BMI below 85% at diagnosis or one of the alternative inclusion criteria listed. Alternative inclusion criteria include any of the following: diabetes diagnosis <12 months of age, diagnosis of renal disease, liver disease, pancreatic exocrine deficiency, chronic diarrhea, genitourinary malformations, gout, autism, adrenal insufficiency, structural heart defects, deafness, diabetes insipidus, cerebellar hypoplasia, epilepsy, neonatal hypoglycemia, aniridia, optic atrophy retinal dystrophy, cataracts, pigmented hypertrichosis, hypoparathyroidism, or a prepubertal presentation of type 2 diabetes mellitus (T2DM) [1]. After consultation at the CHOP Center for Atypical and Monogenic Diabetes, genetic testing for monogenic forms of diabetes was completed at a CLIA‐certified lab. The majority of the subjects had genetic testing at the University of Pennsylvania Genetic Diagnostic Laboratory. Genomic DNA was isolated from peripheral blood lymphocytes of the tested individual and used as a template for multiplex PCR amplification and next‐generation sequencing (NGS) of all the coding exons and flanking intron (+/‐20bp) sequences of the ABCC8, AKT2, BLK, CEL, GCK, HNF1A, HNF1B, HNF4A, INS, INSR, KCNJ11, KLF11, NEUROD1, PAX4, and PDX1 genes. The screen for mutations using NGS was performed on Illumina Miseq or Nextseq platform. The limit of variant allele detection is 20% at 200x coverage. The threshold for mutation detection is set at 20x reads without strand bias. Confirmation of mutations was performed by repeated sequencing of independent replicate sample on the same platform. Known variants with minor allele frequencies (MAFs) greater than 1% in the Exome Aggregation Consortium are filtered out of the analysis. The limit of variant allele detection is 10% at 200x coverage. The threshold for mutation detection is set at 20x reads without strand bias. Regions with low coverage were Sanger sequenced for analysis.

Subjects with suspected monogenic forms of diabetes were enrolled in the Atypical and Monogenic Diabetes Registry after written consent and assent were obtained. This study was approved by the Institutional Review Board of the Children’s Hospital of Philadelphia (IRB# 15‐0283).

### 2.2. Chart Extraction

Once enrolled in the Atypical and Monogenic Diabetes Registry, clinical data, including genetic testing results, were extracted from the medical record. Extracted data included age, height, weight, BMI, and laboratory values at the time of diagnosis, past medical history, birth history, family history, diabetes treatment regimen, additional laboratory testing both prior to and after diabetes diagnosis, and radiology results including renal ultrasound, abdominal ultrasound, pelvic ultrasound, and pituitary MRI. In addition, a history of autism, developmental delays, and symptoms of social pragmatic and communication disorder were extracted from the medical record as well as collected from direct interviews with subjects and parents/guardians. Clinical data were extracted annually.

### 2.3. Clinical Definitions

The diagnosis of diabetes mellitus was made based on the criteria established by the American Diabetes Association, [10] defined as fasting glucose ≥126 mg/dL (≥6.99 mmol/L), hemoglobin A1C ≥6.5%, or random glucose ≥200 mg/dL (≥11.1 mmol/L) with symptoms. BMI percentile for age and sex was extracted from the medical record based on 2002 CDC growth charts [11]. Serum transaminases were characterized as abnormal if they were greater than or equal to two times the upper limit of pediatric reference ranges. Classification of fasting serum lipid concentrations was determined based on pediatric guidelines established by the National Heart, Lung, and Blood Institute (NHBLI), and endorsed by the American Academy of Pediatrics, the American Heart Association, and the American College of Cardiology. Specifically, the NHBLI defines borderline high lipid values for children as triglycerides >90 mg/dL (>1.02 mmol/L), LDL‐C >110 mg/dL (>2.85 mmol/L) HDL‐C <45 (<1.17 mmol/L), and non‐HDL cholesterol >120 mg/dL (>3.11 mmol/L), and high values as triglycerides >130 mg/dL (>1.47 mmol/L), LDL‐C >130 mg/dL (>3.37 mmol/L), and HDL‐C <40 (<1.04 mmol/L) and non‐HDL cholesterol >145 mg/dL (>3.76 mmol/L) [12]. Hyperparathyroidism was defined as a PTH >65 pg/mL (>65 ng/L) with calcium that falls within the reference range based on clinical consensus and the institution’s laboratory reference ranges for age [13]. All PTH and calcium testing were performed at the CHOP clinical laboratory using consistent reference ranges.

### 2.4. Statistical Analysis

Welch’s Analysis of Variance (ANOVA) and Mann–Whitney *U* tests were used to identify statistically significant differences in clinical variables between MODY 5 and pediatric T1DM and T2DM reference groups using a statistical threshold of *p* <  0.05 to define significance. Spearman’s rank correlation coefficient was used to evaluate the relationship between triglycerides and hemoglobin A1c measurements. Clinical data from the standardized diabetes SEARCH cohort was used to define the pediatric Type 1 DM and Type 2 DM reference groups, acknowledging that the SEARCH study did not prescreen individuals to remove cases of undiagnosed monogenic diabetes [14–16]. STATA 16.1 and GraphPad Prism 7.0 were used to perform statistical analyses.

## 3. Results

Between 2013 and 2022, a total of 11 subjects were identified with *HNF1B*‐related disease who were enrolled in the Children’s Hospital of Philadelphia Atypical Diabetes Registry. Individuals identified with a *HNF1B* mutation were between ages 4–18 years and 6 of the 11 individuals were female (Table 2). Five (45%) of the cases were from pathogenic mutations altering a single base pair in *HNF1B*, and the other 6 (55%) were 17q12 microdeletions resulting in *HNF1B* haploinsufficiency, which is similar to the distribution reported in other cohorts of *HNF1B*‐disease [17, 18]. Over two‐thirds of the subjects had a birthweight characterized as small for gestational age (birth weight <10%ile for gestational age). Nine of 11 subjects had a BMI <85^th^ percentile for age at the time of diagnosis with an average BMI Z‐score of −0.4 ± 1.58. Regarding endocrinological manifestations, 82% had a diagnosis of diabetes mellitus at the time that the *HNF1B* mutations or deletion was identified, 91% had abnormal lipid profiles following diagnosis of *HNF1B*‐related disease based on peak fasting level, and 55% had evidence of hyperparathyroidism based on peak PTH and corresponding serum calcium level. Renal disease in subjects with *HNF1B* mutations included cystic kidney disease, mild renal dysplasia, and chronic kidney disease requiring renal transplant. Six of the 11 subjects (55%) had a diagnosis of chronic kidney disease, one who received a kidney/liver transplant (subject 6) and another subject who is currently listed for renal transplant (subject 7) (18%). Additionally, four subjects (36%) have serum magnesium levels <1.2 mg/dL and were prescribed supplementation for hypomagnesemia, and one subject is currently on treatment for hyperuricemia. Five subjects (45%) had a history of liver dysfunction; two with a history of neonatal cholestasis, and three with persistently elevated transaminases (ALT, AST, GGT), including the subject who received the combined kidney/liver transplant (subject 6). Only two subjects had known pancreatic exocrine insufficiency, characterized by low fecal elastase measurements and decreased levels of fat‐soluble vitamins along with complaints of abdominal pain and diarrhea. Symptoms improved after treatment with pancreatic enzymes replacement at meals. Of the female patients in the cohort, five had normal pelvic ultrasounds. The sixth female in the cohort did not have a pelvic ultrasound but reported regular monthly menses. Autism spectrum disorder was diagnosed in four subjects (36%) (three with a 17q12 deletion and one with a *HNF1B* single base mutation), and there were five additional subjects or parents reporting symptoms consistent with social pragmatic and communication disorder.

**Table 2:** Patient Demographics.

Patient	Age at diagnosis	Current age	Sex	Type of mutation	Birth weight	BMI at diagnosis	Diabetes mellitus	Abnormal lipids	Hyper‐PTH	Other medical problems
1	12 (2013)	21	M	*HNF1B* c.494G >A p.Arg165His	SGA	15.81 (9%)	Yes	Yes	No	Renal dysplasia with CKD
										Hepatitis
										Pancreatic exocrine insufficiency

2	14 (2014)	20	F	17q12 del	AGA	19.92 (54%)	Yes	Yes	No	Cerebellar atrophy
										*s*/*p* CVA ASD/developmental delay

3	9 (2015)	14	F	17q12 del	SGA	19.25 (85%)	Yes	Yes	Yes	Cystic kidney disease
										Hypertension
										Conjugated hyperbilirubinemia
										ASD/speech delay

4	4 (2015)	10	F	*HNF1B* c.541C >T p.Arg181Ter	LGA	14.55 (26%)	No	No	No	Prematurity

5	17 (2016)	22	F	17q12 del	SGA	16.52 (1%)	Yes	Yes	No	Renal dysplasia

6	12 (2017)	15	F	17q12 del	SGA	16.93 (30%)	Yes	Yes	Yes	Renal dysplasia with CKD
										Hypertension
										Chronic cholestasis

7	12 (2018)	14	M	*HNF1B* c.1006delC p.His336ThrfsX40	AGA	32.23 (99%)	Yes	Yes	Yes	Growth hormone deficiency (GHSR mutation)
										Renal dysplasia with CKD
										ASD/developmental delay
										Anxiety/OCD/Tourette’s

8	16 (2018)	18	F	*HNF1B* point mutation	SGA	19.18 (31%)	Yes	Yes	Yes	Congenital panhypopituitarism
										Ectopic pituitary
										Cystic kidney disease/CKD

9	15 (2019)	17	M	*HNF1B* c.1431G >C p.Gln477His	SGA	20.66 (53%)	No	Yes	Yes	GNAS mutation
										Hypothyroidism
										Renal dysplasia with CKD

10	18 (2020)	20	M	17q12 del	SGA	27.7 (92%)	Yes	Yes	Yes	Renal dysplasia with CKD
										Hypertension
										ASD/developmental delay

11	17 (2021)	18	M	17q12 del	SGA	16.02 (0.1%)	Yes	Yes	No	Pelviectasis of kidney elevated liver enzymes pancreatic exocrine insufficiency

Summary statistics	13.3 ± 4.1	17.2 ± 3.6	45% Male	45% Heterozygous mutation	72% SGA	BMI Z‐score‐0.40 ± 1.58	82%	91%	56%	91% Renal disease
										45% Liver dysfunction
										36% Autism spectrum disorder
			55% Female	55% Deletion						

### 3.1. Glucose Homeostasis

Seven subjects in our cohort (64%) were diagnosed with diabetes mellitus as the initial endocrine‐related medical condition and were subsequently found to have an *HNF1B* mutation after the diabetes diagnosis. Of the remaining four subjects, one was diagnosed based on family history and three during a genetic workup secondary to renal disease. Of the four subjects who were found to have *HNF1B* mutations at the time of enrollment, two subsequently developed diabetes mellitus. As seen in Table 3, most subjects were diagnosed with diabetes in early adolescence and were pubertal at the time of diagnosis, with the mean age of diagnosis of 14.2 years (range 9–18 years). Only one subject in this cohort presented with diabetic ketoacidosis (DKA) and only three presented with diabetic ketosis, despite significant hyperglycemia. The average plasma glucose at the time of diagnosis was 506 ± 433 mg/dL (range 214–1520 mg/dL) [28.1 ± 24.0; range 11.9–84.4 mmol/L], and the average hemoglobin A1c at diagnosis was 10.2% ± 3.7 (range 6.1 to >14%) [88 ± 17; range 43 to >130 mmol/mol). Four subjects presented with an HbA1C >13% [>119 mmol/mol] highlighting that the hyperglycemia was profound and likely slowly progressed over several months. In addition, serum c‐peptide concentration was elevated at diagnosis in five patients (45%), ranging from 0.2 to 19.1 with an average of 5.6 ± 6.6 ng/mL [1.9 ± 2.2; range 0.07–6.3 nmol/L]. When compared to the SEARCH cohort of individuals with type 1 diabetes mellitus who presented without DKA, patients with *HNF1B* diabetes without DKA presented on average at an older age (13.8 years versus 9.4 years; *p* =  0.005), and with a higher average c‐peptide (6.3 ng/mL [2.1 nmol/L] versus 0.5 ng/mL[0.17 nmol/L]). Although the average hemoglobin A1c was higher in the CHOP *HNF1B* diabetes cohort, this was not significantly different from the SEARCH Type 1 cohort (9.8% versus 8.5%; *p* =  0.34) [15].

**Table 3:** Glucose Homeostasis.

Patient	Initial presentation of diabetes mellitus					Current treatment		
	Known *HNF1B* mutation	Age of onset	Presentation	HbA1c at diagnosis (%)	C‐Peptide (ng/mL)	Recent HbA1C (%)	Treatment	TDD (U/kg/day)
1	No	12	Hyperglycemia	6.8	2.98	8.8	Basal bolus	0.44
2	No	14	Ketosis	10.09	2.77	6.5	Basal bolus	0.31
3	No	9	Hyperglycemia	13.6	1.72	8.2	Basal bolus	0.67

4	Yes	No known diabetes mellitus						

5	No	17	Ketosis	>14	0.6	7.2	Basal bolus	0.9
6	No	12	Hyperglycemia	7.0	9.5	6.1	Basal bolus	0.96
7	Yes	12	Hyperglycemia	6.1	19.1	6.3	Basal only	0.05
8	Yes	17	Hyperglycemia	6.5	12.6	6.4	Lifestyle modifications	—

9	Yes	No known diabetes mellitus						

10	No	18	Diabetic ketoacidosis	>14%	0.2	5.2	Basal bolus	0.77
11	No	17	Ketosis	>14	1.0	5.2	Basal bolus	0.79

HNF1B summary statistics:	4/11 HNF1B mutation known prior to diabetes onset	14.2 years ±3.2 (9–18)	1/11 DKA	10.2% ± 3.7 (6.1–14)	5.6 ± 6.6 (0.2–19.1)	6.7% ± 1.2 (5.2–8.8)	8/11 Basal bolus insulin	0.61 ± 0.32 (0.05–0.96)
			3/11 Ketosis					
			5/11 Hyperglycemia	Without DKA: 9.8% ± 3.6	Without DKA: 6.3 ± 6.7			
			2/11 No known DM					

SEARCH T1DM Cohort *w*/*o* DKA	N (A)	9.4 c 4.1 *p* = 0.005	—	8.5% ± 1.4 *p* = 0.34	0.5 (0.2–1)	—		

*Note*: HNF1B summary statistics: number of individuals affected/total or mean ± 95% CI (range).

All subjects initially diagnosed with diabetes mellitus prior to the identification of an *HNF1B* mutation were treated with a basal/bolus insulin regimen, either by subcutaneous injection or insulin pump. One subject was initially treated with a sulfonylurea after *HNF1B* diabetes diagnosis, but he experienced declining renal function, with his chronic kidney disease progressing from stage 2 to stage 4, along with a rising HbA1c necessitating discontinuation of the sulfonylurea and re‐initiation of basal/bolus insulin therapy. Of the two subjects with known *HNF1B* mutations who developed diabetes following the identification of the mutation, one required initiation of basal insulin due to worsening hyperglycemia and the other has been able to treat hyperglycemia through lifestyle and dietary modifications. During the study period, the average HbA1C for *HNF1B*‐monogenic diabetes patients was 6.7 ± 1.2% (range 5.2–8.8%) with an average insulin requirement of 0.61 ± 0.32 u/kg/day (range 0.05–0.96 u/kg/day) indicating relatively good glycemic control.

### 3.2. Lipid Profiles

Ten of the 11 subjects with *HNF1B*‐related disease (91%) had abnormal lipid profiles with eight of the 11 subjects (73%) meeting the criteria for diagnosis of dyslipidemia based on established pediatric guidelines from the NHBLI [12]. As seen in Table 4, lipid profiles demonstrated: 7/11 with non‐HDL cholesterol >120 mg/dL (>3.11 mmol/L), 10/11 with triglycerides >90 mg/dL (>1.02 mmol/L), 5/11 with LDL‐C >110 mg/dL (>2.85 mmol/L) and 5/11 with HDL‐C < 45 (< 1.17 mmol/L). Of these, several individuals had severely elevated triglycerides and one had extreme elevations in all lipid parameters. When compared to SEARCH cohorts for both type 1 diabetes and type 2 diabetes, the individuals with *HNF1B* diabetes had higher fasting triglyceride and LDL‐C concentrations, as well as higher HDL levels. Mean triglycerides levels in individuals with *HNF1B* diabetes were 224 mg/dL (2.53 mmol/L) compared to 72.6 mg/dL (0.82 mmol/L) in T1DM and 137.4 mg/dL (1.55 mmol/L) in T2DM; *p* <  0.05; mean LDL‐C concentrations were 128 mg/dL (3.31 mmol/L) compared to 93 mg/dL (2.41 mmol/L) in T1DM and 100.9 mg/dL (2.61 mmol/L) in T2DM; *p* <  0.05 [19]. Cholesterol levels were assayed in the setting of well‐controlled diabetes (average HbA1c 6.7%), and there was no direct correlation between triglycerides and HbA1c (*r* = 0.14, *p* = 0.72).

**Table 4:** Lipid Profiles.

Patient	Non‐HDL‐C (mg/dL)	Triglycerides (mg/dL)	LDL‐C (mg/dL)	HDL‐C (mg/dL)	HbA1C ^∗^ (%)	Creatinine ^∗^ (mg/dL)	Management
1	172	162	140	55	8.8	1.46	Dietary changes
2	176	741 ^∗∗^	86	34	5.9	0.6	Dietary changes
3	104	116	81	71 ^∗∗^	6.7	0.5	Dietary changes
4	106	78	90	54	5.2	0.69	None
5	128	121	104	51	7.2	0.56	None
6	301	103	281 ^∗∗^	149 ^∗∗^	6.2	1.2	Atorvastatin
7	286	493 ^∗∗^	185 ^∗∗^	37	6.3	2.74	Atorvastatin
8	106	142	78	70 ^∗∗^	6.4	1.84	Dietary changes
9	99	136	72	32	5.5	1.1	Dietary changes
10	199	255	148	41	5.4	2.6	Dietary changes
11	167	113	144	42	5.2	0.84	Dietary changes
HNF1B summary statistics	168 ± 71	224 ± 207	128 ± 63	58 ± 33	6.3 ± 1.1	1.28 ± 0.80	—
NHLBI pediatric lipid normal ranges	<120	<90	<110	<45	—	—	—
SEARCH Type 1 cohort	—	72.6 ± 59.7	93 ± 26.3	51.8 ± 12.2	—	—	—
SEARCH Type 2 cohort	—	137.4 ± 93.6	100.9 ± 28.4	41 ± 9.4	—	—	—
*p*‐value	—	*p* = 0.0011	*p* = 0.02	*p* = 0.0002	—	—	—

*Note*: Summary statistics: SEARCH Type 1 cohort and SEARCH Type 2 cohort: mean ± 95% CI.

^∗^Creatinine and HbA1C at time of peak cholesterol.

^∗∗^Severely elevated.

All subjects with dyslipidemia were counseled on dietary changes by a registered dietician, and three individuals were prescribed statin therapy. Of note, the subjects who started on adjunct therapy for hyperlipidemia were also taking psychiatric medications including aripiprazole previously reported to be associated with hypertriglyceridemia [20].

### 3.3. Calcium Homeostasis

Despite having high‐normal serum calcium concentrations (mean 9.9 mg/dL [2.47 mmol/L], range 9.4–10.3 mg/dL [2.35–2.58 mmol/L]), 6/11 patients also had a history of elevated PTH (>65 pg/mL or ng/L) consistent with the diagnosis of hyperparathyroidism (Table 5). All but two patients had normal serum creatinine values (mean 0.91 mg/dL [80.5 μmol/L], range 0.6–1.6 mg/dL [45.8–122 μmol/L]), indicating that for the majority of subjects, the elevated PTH preceded the decline in kidney function, consistent with a diagnosis of primary hyperparathyroidism. The majority of patients with primary hyperparathyroidism were not deficient in vitamin D (vitamin D 25‐hydroxy: mean 33 ng/mL [82 nmol/L], range 10.8–69.2 ng/mL [27–173 nmol/L]). Seventy‐eight percent of the subjects had serum magnesium concentrations that were less than 1.8 mg/dL (0.74 mmol/L), a finding that has been attributed to renal magnesium wasting and is potentially a biomarker for the presence of *HNF1B* renal disease. Subject 7 developed worsening kidney disease and eventually presented with hypercalcemia secondary to hyperparathyroidism and has been successfully treated with cinacalcet, which functions to directly lower PTH concentrations by increasing the sensitivity of the calcium‐sensing receptor, resulting in inhibition of PTH secretion.

**Table 5:** Calcium Homeostasis.

Patient	Peak PTH (pg/mL)	Calcium (mg/dL)	Phosphorous (mg/dL)	Magnesium (mg/dL)	Creatinine ^∗^ (mg/dL)	Vitamin D 25‐OH (ng/mL)	Current management
1	52	9.6	2.6	1.3	1.23	21.1	Cholecalciferol
2	20	9.9	3.0	1.4	0.75	27.9	None
3	95.5	10.2	4.2	1.4	0.6	24.2	None
4	51.5	10.2	5.2	—	0.4	50.9	None
5	14	9.9	2.9	1.1	0.56	—	Magnesium
6	312	10.3	5.3	2.0	1.6	14.7	Calcitriol, Ca carbonate
7	265	9.4	8.1	2.2	0.7	69.2	Cinacalcet
8	364	10.3	5.3	—	0.9	25.6	Calcitriol, cholecalciferol
9	146	9.8	3.5	1.6	1.0	40.1	None
10	112	9.8	5.2	1.6	1.6	45.1	Cholecalciferol
11	34	9.9	3.6	1.1	0.68	10.8	Cholecalciferol
Summary statistics	133.3 ± 124.4	9.9 ± 0.29	4.4 ± 1.6	1.5 ± 0.4	0.91 ± 0.41	33.0 ± 18.1	—

^∗^Creatinine at time of Peak PTH.

## 4. Comparison to Other Pediatric *HNF1B* Cohorts

Table 6 summarizes data from five recently reported cohorts of individuals with *HNF1B* disease that included pediatric subjects (2009–2022) and one meta‐analysis from 2020. Cohorts described by Warncke et al. [3] and Sztromwasser et al. [21] were recruited from diabetes patient registries similar to the CHOP *HNF1B* disease cohort. Individuals with *HNF1B* diabetes described by Ge et al. [22] were identified through a meta‐analysis of the literature and included both pediatric and young adult cases and due to the meta‐analysis format may include a limited number of subjects described in the other cohorts. Patients with *HNF1B* disease described by Nagano et al.. [23], Lim et al. [24], and Adalat et al. [25] were identified from registries of individuals with renal malformations or dysfunction. Warncke et al. [3] describe a cohort of 35 children and adolescents with *HNF1B* diabetes identified from a multicounty diabetes registry (DPV) in Europe containing over 75,000 individuals. Individuals in the Warncke cohort had pancreatic *β*‐cell antibodies assessed and the diagnosis of *HNF1B* diabetes was made by local clinicians and confirmed with genetic testing. The mean age of diabetes diagnosis (13.5 years) was similar to the CHOP cohort (14.2 years) and the BMI SDS score was not elevated in either cohort (0.6 vs −0.4). The average HbA1C and c‐peptide levels at diagnosis were much lower in the Warncke cohort compared to CHOP (7.0% and 1.5 ng/mL vs., 10.2% and 5.6 ng/mL) but a similar percentage of individuals were treated with insulin (65.2% vs., 72%) with similar total daily insulin doses (0.7 vs., 0.61 units/kg/day). Hyperlipidemia and extrapancreatic conditions were common in both groups. Ge et al. [22] performed a meta‐analysis of literature describing individuals with MODY5 or *HNF1B* diabetes, and using 48 separate publications and 61 total cases, 34 of which had available data regarding the clinical diabetes presentation. Although specific data on individuals diagnosed with *HNF1B* diabetes prior to age 18 was not available, the median age of diagnosis was 16, with 15% of cases presented prior to age 10 years (*n* = 5 cases). The mean HbA1C was 9.4% and 15% of individuals presented with DKA, higher than any other cohort. Pancreatic dysgenesis, renal cysts and dysplasia, and hypomagnesemia were very common in this cohort.

**Table 6:** Pediatric *HNF1B* Diabetes Cohorts.

Publication: First author, Journal, Year, PMID	Cohort description	# cases of *HNF1B* diabetes diagnosed before age 18	Mean age, HbA1C, and c‐peptide at pediatric *HNF1B* diabetes diagnosis	Pediatric diabetes treatment	Pediatric BMI SDS at *HNF1B* diabetes diagnosis	Pediatric extra‐pancreatic symptoms	Conclusions
Nagano, Clinical Exp Nephrol, 2019, PMID: 31131422	• Recruitment from individuals with renal abnormalities without known genetic mutations. • 2010–2018 • 19 *HNF1B* heterozygous variants and 14 17q12 deletions (total *n* = 33) • Pediatric cases: 18/33	• Two subjects diagnosed with diabetes before age 18 (ages 10 and 14), both with heterozygous variants	• NR	• NR	• NR	• Neurological symptoms: 5/33. • One with autism (age 10) • All patients with neurological abnormalities had 17q12 deletions	• The median age at *HNF1B* disease diagnosis was less for those with heterozygous variants (11 years) than 17q12 deletions (32 years) • eGFR was significantly lower in heterozygous variants vs. deletions (~38 vs., ~ 50 mL/min/1.73m^2^)

Warncke, J Clin Endocrinol Metab, 2019, PMID: 30535056	• DPV Diabetes Registry. • 76,836 individuals < 20 years old with diabetes. • 1995–2017 • *HNF1B* diabetes was diagnosed by local clinician and confirmed with genetic testing. • Pancreatic *β*‐cell antibodies were measured.	• 35 pediatric subjects diagnosed with *HNF1B* diabetes (16 with heterozygous variants; 19 with 17q12 deletion)	• Age: 13.5 years (11.2–15.7) • HbA1C: 7.0 % (6.0–12.6) • C‐peptide: 1.5 ng (mL) (±0.5)	• Insulin: 67% • Mean insulin dose: 0.7 u/kg/day (±0.1)	• 0.6 (−0.3 to 1.4)	• Dyslipidemia: Percentage of HNF1B diabetes subjects with dyslipidemia was similar to T2D (65% vs., 68.8%) Extrapancreatic symptoms in 40% of individuals (mostly renal)	• The DPV Pediatric Diabetes Registry allowed for precise characterization of the diabetes phenotype and treatment of MODY cases • 19/35 patients with *HNF1B* diabetes were originally diagnosed with T1D or T2D and were reclassified as HNF1B diabetes after an average of 1.6 years

Adalat, J Am Soc Nephrol, 2009, PMID: 19389850	• *HNF1B* genetic testing performed on 91 individuals with renal malformations. • 2000–2007 • Identified 21 heterozygous variants and 12 deletions (*n* = 3 total) • Pediatric cases: 21/33	• Five subjects with *HNF1B* diabetes diagnosed before 18 years	• Age: 12 (10–14) • HbA1C: NR • C‐peptide: NR	• NR	• NR	• NR	• All individuals with *HNF1B* disease had abnormal renal findings on fetal ultrasound • Low serum Mg may be a biomarker to identify patients with *HNF1B* renal disease

Sztromwasser, Pediatric Diabetes, 2020, PMID:31825128	• Polish Monogenic Diabetes Registry (*n* = 36) • 2005‐–2018 • 15 individuals identified with *HNF1B* variants or deletions: 7 adults, 8 children.	• 12/15 individuals with *HNF1B* mutations were diagnosed with IGT or diabetes • Unclear how many were children at time of diagnosis • Two individuals with *HNF1B* diabetes had positive pancreatic *β*‐cell antibodies • Whole *HNF1B* gene deletions were identified in 8/15 individuals	• Data on Pediatric *HNF1B* diabetes cases not presented separately. • Overall age: 16.3 years (13.0–25.4) • HbA1C: 7.9 % (7.4–8.7) • None with DKA	• NR	• NR	• Overrepresented conditions in *HNF1B* diabetes mutations carriers: 1) pancreatic abnormalities (atrophy, PEI calcifications; cystic kidneys, elevated liver enzymes	• Presence of IGT and kidney disease in proband and one parent is highly predictive of *HNF1B* mutations • Unclear how many of the 12 with *HNF1B* diabetes were diagnosed as children

Lim, J Clin Med, 2020, PMID: 32708349	• 110 individuals with pediatric renal disease without a known genetic mutation were screened for *HNF1B* mutations at a single pediatric medical center in Korea. • 2008–2019 • 14 individuals with HNF1B mutations were identified (6 gene deletion, 8 heterozygous mutations)	• Five were diagnosed with *HNF1B* diabetes before age 18 (1 case diagnosed after renal transplant)	• 14.6 (IQR: 11.6–16.5)	• Insulin and oral hypoglycemics reported	• NR	• 29% with pancreatic hypoplasia or cysts • 57% liver dysfunction • 57% Hyper‐PTH • 50% hypokalemia • 21% hypomagnesemia • 79% hyperuricemia • 21% had neurological deficits	• Diagnosis of *HNF1B* mutations is clinically difficult due to extreme phenotypic variability and incomplete penetrance • Some phenotypes develop with age • Pancreatic abnormalities were more common in individuals with missense mutations in this cohort • Neurological deficits were identified in both individuals with gene deletions and heterozygous mutations • Genotype did not correlate with renal dysfunction

Ge, Frontiers in Endocrinology, 2020, PMID: 35846334	• Meta‐analysis of 48 manuscripts containing search terms related to MODY 5 or *HNF1B* diabetes up until 2022. • Identified 61 subjects. • Diabetes characteristics available on 39 subjects. • Individual references not provided so may include individuals reported in other cohorts.	• # of individuals diagnosed with diabetes before age 18 not provided	• Median age of diabetes diagnosis was 16.0 years • 30/34 (84%) were diagnosed with *HNF1B* diabetes < 25 years • 15% were diagnosed with *HFN1B* diabetes before age 10 years • Mean HbA1C 9.4% • Mean c‐peptide: 1.24 ng (mL) • 16% presented with DKA	• 80% treated with insulin • 10% oral hypoglycemics • 2% GLP‐1	BMI SDS not available:38% BMI < 18.5; 54% BMI 18.5‐–25; 7% BMI >25	• 92% hypomagnesemia • 72% renal cysts • 19% renal dysplasia • 72% pancreatic dysplasia	• Adolescent/early adult onset of diabetes with low or normal BMI, renal cysts, Hypomagnesemia, and pancreatic dysplasia should be considered for *HNF1B* diabetes testing • At diagnosis, most *HNF1B* diabetes patients were diagnosed with T1D or T2D • *HNF1B* diabetes most commonly present in later adolescence or young adults but can present before age 10 years • Renal abnormalities were common • Hypomagnesemia differentiated individuals with *HNF1B* disease and other genetic forms of renal cysts and dysplasia

Craven	• 11 individuals with pediatric *HNF1B* disease at a single pediatric tertiary care center in Philadelphia (PA) • Utilized a diagnostic algorithm to identify children with atypical/monogenic diabetes based on negative *β*‐cell antibodies and other clinical features or history of *HNF1B* mutation.	• Nine subjects with *HNF1B* diabetes diagnosed before 18 years • 33% with *HNF1B* heterozygous mutation and 67% with *HNF1B* deletion	• Age: 14.2 years (9–18) • HbA1C: 10.2% (6.1–>14%) • 1 with DKA; 3 with ketones	• Insulin: 73% • Mean insulin dose: 0.61 u/kg/day	−0.40 (± 1.43)	• 72% SGA BW • 91% Dyslipidemia • 86% Hyper‐PTH • 91% renal disease • 45% Liver dysfunction • 36% autism spectrum disorder	• Well‐phenotyped case series of pediatric *HNF1B* disease focusing on diabetes and endocrine features • Majority of subjects with *HNF1B* diabetes had clinical features that overlapped with T1D and T2D • Dyslipidemia, hyperparathyroidism, and renal disease were common • ASD was identified in individuals with both gene deletion and heterozygous mutations

Abbreviation: NR, not reported.

The description of *HNF1B* pediatric diabetes cases described in the publications by Warncke, Ge and at CHOP had diabetes characteristics that overlapped T1D and T2D likely due to the fact that the diabetes phenotype includes evidence of both relative insulin deficiency and insulin resistance. The Warncke *HNF1B* pediatric diabetes cohort had more similarities in their diabetes presentation to the pediatric T2D patients in their own registry based on age of diabetes diagnosis, HbA1C, c‐peptide at diagnosis, and presence of hyperlipidemia. The Ge cohort had a higher average HbA1C at presentation than the other groups including a large number of individuals presenting in DKA. The CHOP *HNF1B* diabetes subjects had higher average HbA1C, c‐peptide, and lipid profile components compared to both T1D and T2D SEARCH data.

The cohort described by Sztromwasser et al. [21] included 15 individuals with *HNF1B* disease (7 adults and 8 children), 12 of which had impaired glucose tolerance (IGT) or diabetes with the average HbA1C at diagnosis of 7.9%. The number of individuals diagnosed with *HNF1B* diabetes prior to age 18 was not identified but the average age of diabetes diagnosis was 16.3 years, which is older than the other cohorts. Extra pancreatic symptoms were common in this cohort as well.

The cohorts described by Nagano et al., Lim et al., and Adalat et al. were identified from larger registries of patients previously diagnosed with renal disease [23–25]. There are only a total of 12 additional cases of pediatric *HNF1B* diabetes described in these cohorts, and there is limited data presented describing the clinical diabetes characteristics of the individuals in these groups. Extra pancreatic symptoms were highly prevalent. Additionally, similar to the CHOP cohort (Craven), Lim et al. [24] described 8/14 (57%) of the individuals with *HNF1B* disease had hyperparathyroidism. Although secondary and tertiary hyperparathyroidism is common in populations with chronic renal disease, there is evidence in the cohorts of pediatric *HNF1B* disease that the etiology of the hyperparathyroidism may be a primary defect. Studies suggest that *HNF1B* acts as a novel repressor of *PTH* gene transcription thus contributing to the development of hyperparathyroidism [26]. Over half of the CHOP pediatric cohort had evidence of primary hyperparathyroidism, with only one of the six patients having concurrent vitamin D 25‐OH deficiency. Additional *in vitro* studies provide evidence that the transcription factor *HNF1B* plays a role in the development of hyperparathyroidism and is required for PTH activity [26].

All six cohorts included individuals with *HNF1B* heterozygous deletions and *HNF1B* heterozygous mutations, but the prevalence varied based on the population studied. Some groups report mild phenotype/genotype associations including age of diagnosis, eGFR, pancreatic abnormalities, and autism spectrum disease but it is not clear if these relationships persisted throughout all cohorts [23–25]. Additionally, developmental uterine abnormalities have been previously identified in females with *HNF1B* disease [5], but the prevalence of uterine abnormalities in the six cohorts described in Table 2 was very low (<10%). Five of six females in the CHOP cohort had normal pelvic ultrasounds and the sixth patient reported regular periods.

## 5. Conclusion and Recommendations

Pediatric *HNF1B* disease is a rare genetic condition that can be difficult to diagnose based on the extreme phenotypic variability among affected individuals and within families. Although pediatric *HNF1B* diabetes is rare, there are clinical characteristics that are common in these patients that can be used by pediatric endocrinologists to identify candidates for genetic testing. *HNF1B* diabetes often presents in mid‐adolescence with hyperglycemia but the hyperglycemia is not commonly associated with ketosis or acidosis. C‐peptide levels are often measurable despite the degree of hyperglycemia and hyperlipidemia is common. Confirming features including a negative diabetes autoimmune panel, a normal or low (as opposed to elevated) BMI along without evidence of insulin resistance or a prepubertal presentation of diabetes should prompt consideration of monogenic diabetes genetic testing including sequencing and gene deletion analysis. History of IUGR or SGA birthweight and other neonatal diagnoses including hyperglycemia, cholestasis, and renal cysts are commonly reported in patients with *HNF1B* disease. Although pancreatic enzyme insufficiency is found in individuals with *HNF1B* disease, extra pancreatic conditions including renal malformations or dysfunction, hyperparathyroidism, hypomagnesemia, genital tract abnormalities, autism spectrum disorder, and developmental delays are also very common and should prompt consideration of *HNF1B* genetic testing.

The underlying etiology of the increased prevalence of hyperlipidemia in pediatric *HNF1B* diabetes is not known. While factors leading to hyperlipidemia in *HNF1B* disease are unclear, hepatic etiologies such as hepatic insulin resistance and hepatic bile acid abnormalities have been proposed [27]. Patients with *HNF1B* mutations can have neonatal cholestasis and bile duct paucity, as well as reductions in bile acid concentrations and elevated hepatic transaminases (AST, ALT, and GGT) [27]. Mice with liver and biliary‐specific *Hnf1b* inactivation had elevated serum cholesterol and triglycerides levels in a pattern similar to that described in the pediatric *HNF1B* diabetes cohorts suggesting that abnormalities in biliary duct formation and function and bile acid metabolism in subjects with *HNF1B* mutations may lead to hyperlipidemia [28, 29]. Given that over 90% of the individuals in the CHOP *HNF1B* cohort had significant dyslipidemia, elevated lipid markers may be useful as clinical biomarkers to identify pediatric patients who are candidates for *HNF1B* genetic testing in the setting of a negative diabetes autoimmune panel. We suggest repeating the fasting lipid profile 1–3 months after diabetes diagnoses in these individuals and if the lipid markers remain elevated, consider obtaining monogenic diabetes genetic testing for *HNF1B* mutations/deletions.

Most pediatric *HNF1B* diabetes patients require treatment with total daily insulin requirements that are slightly lower than adolescents with T1D. Autism spectrum disorder and developmental delay diagnoses can affect treatment approaches for pediatric patients with *HNF1B* diabetes, especially if daily insulin injections or intensive glucose monitoring are required. Future treatment with GLP‐1R agonists holds promise but more data are needed to assess effectiveness and potential impact on renal function. Islet cell transplant could potentially be considered in patients with *HNF1B* diabetes requiring renal transplant, as there is evidence in the adult *HNF1B* diabetes literature that *β*‐cell function continues to decline over time in subjects with *HNF1B* diabetes [30–32]. Importantly, the potential effects of immunosuppressive therapies on *β*‐cell function and hepatic insulin resistance in the setting of *HNF1B* diabetes are poorly characterized and need to be assessed before this becomes an acceptable approach to treatment [30].

In summary, identifying a molecular diagnosis in pediatric patients with atypical forms of diabetes including *HNF1B* diabetes is important for assessing multisystem disease involvement, counseling regarding heritability, and identifying potential alternative treatment regimens over a lifetime. Additional studies are needed to determine both ideal preventive approaches and optimal treatment plans for *HNF1B*‐related endocrine disorders, including *HNF1B* diabetes but also regarding abnormalities in lipid metabolism and calcium homeostasis which are underreported in the literature.

## Conflicts of Interest

The authors declare no conflicts of interest.

## Author Contributions

M.C. researched the data, wrote the manuscript, and reviewed and edited the manuscript. She has no competing financial interests to declare. V.B. researched the data and reviewed and edited the manuscript. She has no competing financial interests to declare. A.C. researched the data and reviewed and edited the manuscript. He has no competing financial interests to declare. S.E.P. researched the data, wrote the manuscript, and reviewed and edited the manuscript. She has no competing financial interests to declare. S.E.P. is the guarantor for the research described in this manuscript.

## Funding

This publication was supported by the McCabe Foundation (SEP) and the Kahn Foundation (SEP).

## Data Availability

Original data generated and analyzed for this study are included in this published article.
